# Urine and Serum Metabolomics Analyses May Distinguish between Stages of Renal Cell Carcinoma

**DOI:** 10.3390/metabo7010006

**Published:** 2017-02-03

**Authors:** Oluyemi S. Falegan, Mark W. Ball, Rustem A. Shaykhutdinov, Phillip M. Pieroraio, Farshad Farshidfar, Hans J. Vogel, Mohamad E. Allaf, Matthew E. Hyndman

**Affiliations:** 1 Department of Biological Sciences, University of Calgary, Calgary T2N1N4, AB, Canada; osmeduna@ucalgary.ca (O.S.F.); rousha704@gmail.com (R.A.S.); vogel@ucalgary.ca (H.J.V.); 2 James Buchanan Brady Urological Institute, Johns Hopkins Medical Institutions, Baltimore 21287, MD, USA; mark.ball@jhmi.edu (M.W.B.); philpierorazio@gmail.com (P.M.P.); mallaf@jhmi.edu (M.E.A.); 3 Department of Oncology, Cumming School of Medicine, University of Calgary, Calgary, T2N 4N1, AB, Canada; farshidf@ucalgary.ca; 4 Department of Surgery, Cumming School of Medicine, University of Calgary, Calgary, T2N 4N1, AB, Canada; 5 Prostate Cancer Centre, Rockyview Hospital, Calgary T2V 1P9, AB, Canada

**Keywords:** metabolomics, renal cell carcinoma, malignancy, renal lesions

## Abstract

Renal cell carcinoma (RCC) is a heterogeneous disease that is usually asymptomatic until late in the disease. There is an urgent need for RCC specific biomarkers that may be exploited clinically for diagnostic and prognostic purposes. Preoperative fasting urine and serum samples were collected from patients with clinical renal masses and assessed with ^1^H NMR and GCMS (gas chromatography-mass spectrometry) based metabolomics and multivariate statistical analysis. Alterations in levels of glycolytic and tricarboxylic acid (TCA) cycle intermediates were detected in RCC relative to benign masses. Orthogonal Partial Least Square Discriminant Analysis plots discriminated between benign vs. pT1 (R2 = 0.46, Q2 = 0.28; AUC = 0.83), benign vs. pT3 (R2 = 0.58, Q2 = 0.37; AUC = 0.87) for ^1^H NMR-analyzed serum and between benign vs. pT1 (R2 = 0.50, Q2 = 0.37; AUC = 0.83), benign vs. pT3 (R2 = 0.72, Q2 = 0.68, AUC = 0.98) for urine samples. Separation was observed between benign vs. pT3 (R2 = 0.63, Q2 = 0.48; AUC = 0.93), pT1 vs. pT3 (R2 = 0.70, Q2 = 0.54) for GCMS-analyzed serum and between benign vs. pT3 (R2Y = 0.87; Q2 = 0.70; AUC = 0.98) for urine samples. This pilot study suggests that urine and serum metabolomics may be useful in differentiating benign renal tumors from RCC and for staging RCC.

## 1. Introduction

Renal cell carcinoma (RCC) is a metabolic disease that accounts for 5% of all adult malignancies and is the second most lethal urinary cancer after bladder. It is estimated that, in 2016, 62,700 new cases and 14,240 deaths will be recorded in the United States [1,2]. Most renal masses are identified incidentally by cross-sectional imaging, which cannot distinguish RCC from benign renal lesions. Moreover, 20%–30% of small renal masses (SRMs) (<4cm) that are surgically removed are found to be benign, and majority of the resected RCCs in this size range are low grade and thought to be indolent [3]. Therefore, many of these masses are surgically removed or ablated without significant benefit to patients, increasing morbidity and cost to the health care system that could be avoided if accurate, non-invasive diagnosis were possible. Renal mass biopsy may be helpful but is prone to sampling error and ultimately is invasive and associated with some morbidity [4]. Thus; there is no non-invasive means of accurately diagnosing and risk stratifying renal masses.

Altered metabolism is a well-established hallmark of cancer and is directly implicated in the pathogenesis of RCC [5]. Mutations affecting hypoxia inducible factor (HIF), succinate dehydrogenase and fumarate hydratase are known to alter cellular metabolism and contribute to cellular growth [6]. Perturbed glycolysis, TCA cycle, amino acid and fatty acid metabolism have been consistently reported in metabolomics studies as the major metabolic alterations associated with the disease [7], and these direct biochemical changes and the proximity of the renal mass to blood circulation and the urinary collecting system suggest that metabolomics analysis of serum and urine may lead to a quantitative metabolic signature that can distinguish between RCC and benign lesions.

In agreement with this notion, renal cell carcinoma metabolomics has already been applied to tissue [8,9,10], serum [11,12], plasma [13] and urine [14,15] samples. These metabolomics studies were aimed at distinguishing between RCC and disease-free controls and RCC biomarker discovery is already underway. The translation of potential biomarkers from “bench-to-bedside” utility is also vigorously pursued. In more recent studies, NMR analysis distinguished between RCC and controls while demonstrating minimal impact of confounding factors on a 32-metabolite urinary signature [16]. Other authors have identified a 7-metabolite cluster (alanine, creatine, choline, isoleucine, lactate, leucine, and valine) in serum [11], alpha-ketoglutarate and quinolinate in urine [17] and alpha-tocopherol, hippuric acid and myo-inositol in tissue [8], highlighting the diversity in metabolites detected in different biological materials along with the convergence in the overall altered metabolic pathways. 

NMR spectroscopy and chromatography-coupled mass spectrometry (MS) methodologies in combination with multivariate statistical data analysis are currently the most widely employed metabolomics platforms for detecting and measuring metabolites [18]. NMR spectroscopy is directly related to Magnetic Resonance Imaging (MRI), but it identifies and quantitatively measures the concentrations of various compounds [19,20]. Generally, NMR of biofluids is known for its reproducibility, minimal sample preparation requirements and its non-destructive nature [21]. Gas chromatography involves the separation of volatile and semi-volatile compounds, which is then coupled to a mass spectrometer where the metabolites are ionized and resolved per their mass/charge ratio. Gas chromatography-mass spectrometry (GCMS) has a higher sensitivity than NMR spectroscopy but not all features detected can be classified as metabolites. The information obtained from these platforms is complementary [22,23]. Nonetheless, integrating data obtained from complementary metabolomics methods has been shown to provide better model interpretability and improved coverage of the metabolome [24].

In this work, we have applied ^1^H nuclear magnetic resonance (NMR) spectroscopy and gas chromatography mass spectrometry (GCMS) based metabolomics analyses of serum and urine samples with multivariate statistical analysis (individual and combined), in patients undergoing surgical intervention for small renal masses (SRMs) to investigate if metabolic profiles could differentiate between benign and malignant renal masses.

## 2. Results

### 2.1. Samples

Forty (40) malignant samples and thirteen (13) benign samples of serum and urine were assessed. The malignant groups were stratified by pathological stage pT1 (*n* = 28), pT2 (*n* = 1), pT3 (*n* = 10) and pT4 (*n* = 1). The clinicopathologic characteristics of the samples are outlined in Table 1.

### 2.2. ^1^H NMR Spectral Analysis of Serum and Urine Samples

**Figure 1:**
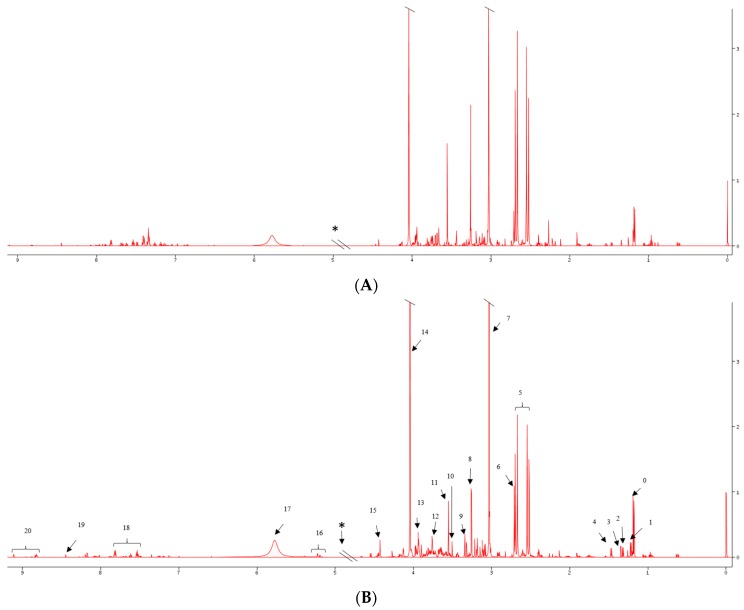
Characteristic NMR spectra of urine samples obtained from (**A**) benign (control); and (**B**) RCC patients 0: 3-aminoisobutyrate, 1: fucose, 2: lactate, 3: threonine, 4: alanine, 5: citrate, 6: dimethylamine, 7: creatinine, 8: trimethylamine, 9: methanol, 10: 3-methylxanthine, 11: glycine 12: gluconate. 13: creatine, 14: creatinine, 15: trigonelline, *—deleted water region, 16: lactose, 17: urea, 18: Hippurate, 19: formate, 20: trigonelline.

For the experiments, 28, 10, 1, 1 and 13 spectra were obtained for the pT1, pT3, pT2, pT4 and benign samples, respectively. Figure 1 shows characteristic NMR spectra of urine samples (A) benign, (B) cancer; visually differential metabolites include increased 3-aminobutyrate, reduced citrate and trimethylamine, reduced glycine and trigonelline in RCC relative to controls. In serum samples, visual inspection of the spectra showed increased glucose, reduced citrate and 3-hydroxybutyrate levels in RCC compared with controls (Supplementary Figure S1).

### 2.3. Distinguishing between Benign and Cancerous Renal Masses in ^1^H NMR and GCMS Datasets

**Figure 2:**
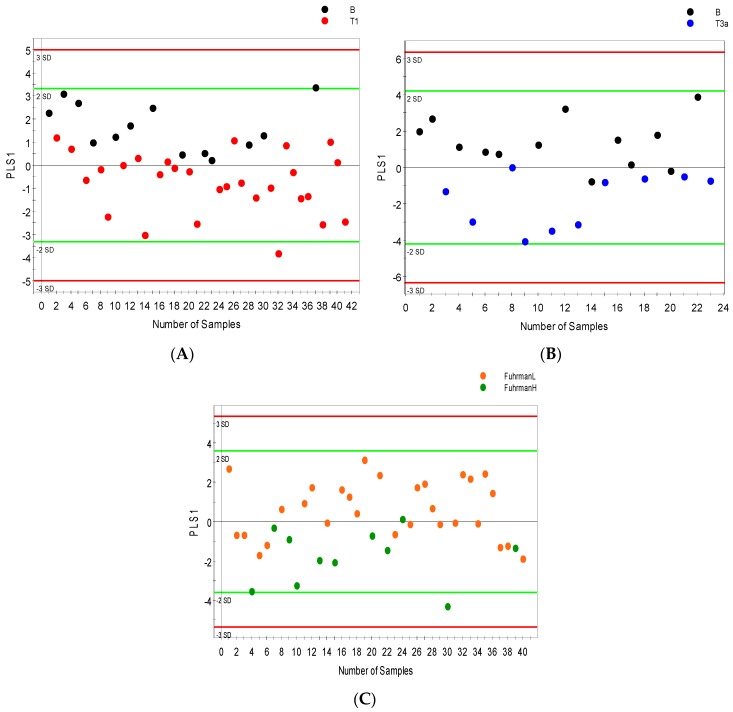

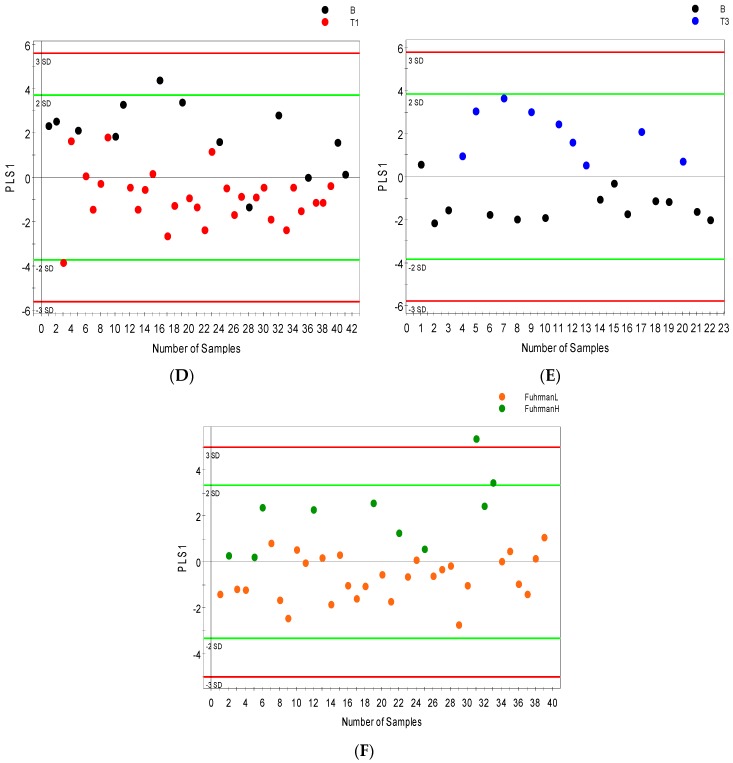
NMR orthogonal partial least squares discriminant analysis (OPLS-DA) models distinguishing between benign and cancer cases. OPLS-DA score scatter plots showing separation in the serum metabolic profile of (**A**) benign versus stage 1 cancer cases; (**B**) benign versus stage 3 cancer cases and (**C**) low grade (FuhrmanL–stages 1 and 2) versus High grade (FuhrmanH–stages 3 and 4) cancer; separation in urine metabolic profile (**D**) benign versus stage 1 cancer cases (**E**); benign versus stage 3 cancer cases (**F**); and low grade (FuhrmanL–stages 1 and 2) versus High grade (FuhrmanH–stages 3 and 4) cancer along their orthogonal partial least squares (OPLS1) and partial least squares components (PLS1).

An average of 56 metabolites were identified and quantified in all serum samples and 70 urine metabolites were detected by ^1^H NMR spectroscopy. The most differential serum metabolites identified based on VIP values > 1 were considered significant. ^1^H NMR spectroscopy mostly distinguished between benign renal lesions and malignant renal masses assigned pathological stage pT1 with minimal overlap between the stages. However, the validation metrics were somewhat poor (R2Y = 0.46; Q2 = 0.28; Figure 2A). Figure 1B also shows some overlap between benign renal lesions and pT3 with improved metrics more typical of a biological system (R2Y = 0.58; Q2 = 0.37; Figure 2B). Low Fuhrman grade (stages 1 and 2) were found to overlap with high Fuhrman grade (stages 3 and 4) (R2Y = 0.37; Q2 = 0.23 Figure 2C). Urine samples analyzed by ^1^H NMR and supervised OPLS-DA (orthogonal partial least squares discriminant analysis) showed improved separation between benign and malignant groups; while the separation between benign and pT1 (R2Y = 0.50; Q2 = 0.37; Figure 2D) was somewhat reduced by overlap between groups and the same is true for the separation between low Fuhrman grade (stage 1 and 2) and high Fuhrman grade (stage 3 and 4) (R2Y = 0.54; Q2 = 0.36 Figure 2F), benign samples were distinguished from pT3 with much better metrics (R2Y = 0.72; Q2 = 0.68; Figure 2E).

**Figure 3:**
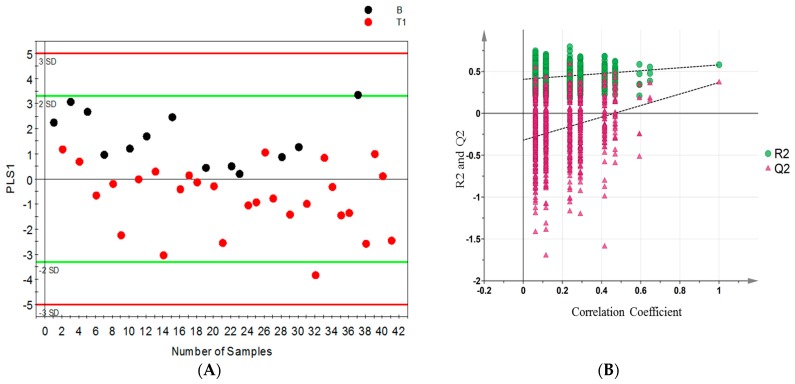
OPLS-DA model differentiating benign (B) from stage 1 (pT1) patients; (**A**) scores scatter plot; and (**B**) permutation plot from 999 permutation tests with Q2 intercept of −0.27 (the more negative the Q2 intercept, the more valid the model).

Permutation tests were carried out to confirm the stability and robustness of the supervised OPLS-DA models presented in this study. Figure 3 shows (A) the scatter score plot and (B) permutation plot of the first model (B vs. pT1) with a Q2 intercept of −0.27, models with negative Q2 intercept are more robust and confirm the stability of the models.

**Figure 4:**
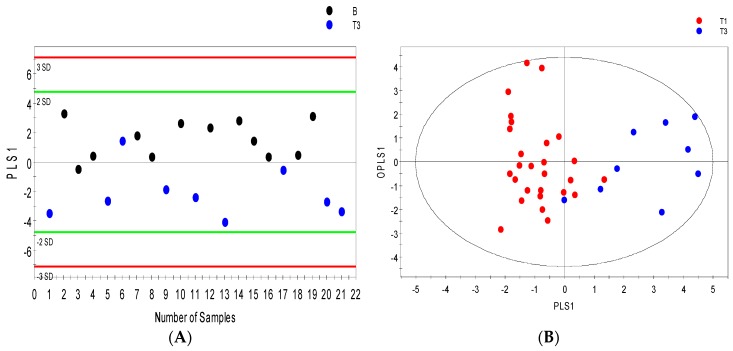

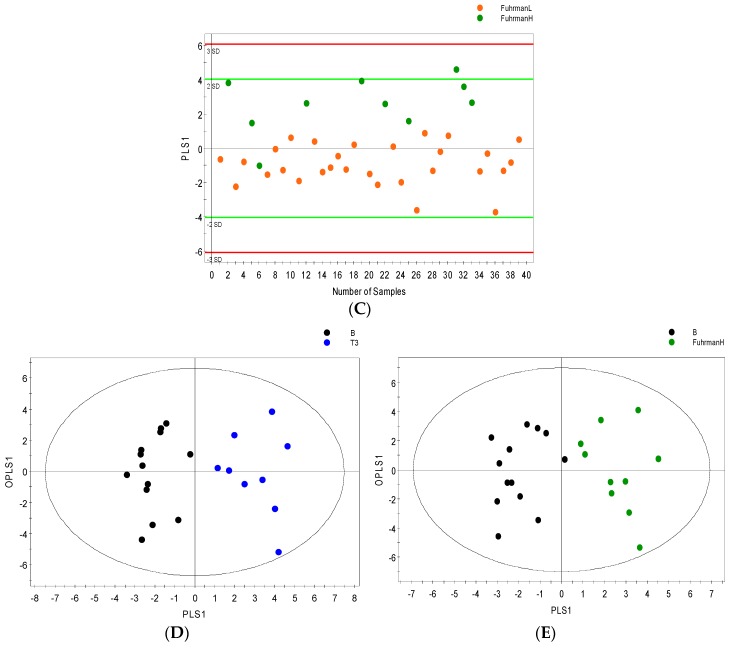
GCMS orthogonal partial least squares discriminant analysis (OPLS-DA) models distinguishing between benign and cancer cases. OPLS-DA score scatter plots showing separation in the serum metabolic profile of (**A**) stage 1 versus stage 3 cancer cases; (**B**) benign versus stage 3 cancer cases; (**C**) low grade (FuhrmanL–stages 1 and 2) versus High grade (FuhrmanH–stages 3 and 4) cancer along their orthogonal partial least squares (OPLS1) and partial least squares components (PLS1); (**D**) OPLS-DA score scatter plots depicting the urine metabolic signature distinguishing benign subjects from stage 3; and (**E**) benign cases from high grade (FuhrmanH–stages 3 and 4), (ellipsoid: Hotelling’s T2, 95% confidence interval).

There were 102 metabolic features detected in the serum profile analyzed by GCMS and up to 200 in urine samples. Only 52 serum features and 58 urine features were identified as known metabolites. After data reduction by relative standard deviation (RSD), metabolites with RSD < 50% were subjected to OPLS-DA analysis. Serum metabolites with VIP values > 1 were considered significant. OPLS-DA distinguished patients with benign renal lesions from those with renal masses assigned pT3 (R2Y = 0.63; Q2 = 0.48; Figure 4A). Furthermore, patients with pT1 disease were separated from those with more advanced RCC pT3 (R2Y = 0.70; Q2 = 0.54; Figure 4B) with minimal overlap between the groups. Figure 4D shows that in GCMS analyzed urine samples, patients with benign renal lesions could be distinctively distinguished from those with pT3 disease (R2Y = 0.87; Q2 = 0.70). Figure 4C,E shows separation between low Fuhrman grade cancer (1 and 2) and high Fuhrman grade cancer (3 and 4) in serum (R2Y = 0.60; Q2 = 0.47) and between benign and high Fuhrman grade urine samples (R2Y = 0.84; Q2 = 0.62), respectively.

### 2.4. Integrative ^1^H NMR and GCMS Data Analysis

The combined dataset consists of 141 serum and 286 urine metabolites/metabolic features, 16 of which are common to both analytic platforms for serum and urine alike. In serum, 58 and 83 metabolites/metabolic features were contributed by ^1^H NMR and GCMS, respectively. Urine, however, had many more metabolites/metabolic features—62 and 224 from NMR and GCMS, respectively. For metabolites that were quantified using both NMR and GCMS platforms, including glutamate, pyruvate, lactate and citrate, the stage-wise trends were consistent. Table 2 shows the statistical metrics and permutation results of all OPLS-DA models, and the more negative the Q2 intercept, the better the validity of the model. The integrated serum OPLS-DA (Supplementary Figure S2A) distinguished patients with benign renal lesions from those with pT1 disease (R2Y = 0.53; Q2 = 0.28). Furthermore, benign patients were distinctively separated from those with renal masses assigned pT3 (R2Y = 0.51; Q2 = 0.40) S2 B. The integrated urine OPLS-DA, showed better separation between benign renal lesions and pT1 disease and between benign and pT3 disease than OPLS-DA models of individually analyzed NMR and GCMS observed (R2Y = 0.94; Q2 = 0.58; R2Y = 0.92; Q2 = 0.73, respectively (Supplementary Figure S2C,D)).

### 2.5. Differential Metabolites

Differential metabolites distinguished benign from cancerous cases and between stages of R CC as identified by the metabolomics platforms used (Table 3). The group of metabolites found to be differential in serum varied considerably from those detected in urine based on individual and integrated data analysis. Nonetheless, glycolytic and TCA cycle intermediates, amino acids and their derivatives were the most significant metabolites identified as potentially useful variables. In serum and urine samples, pyruvate and lactate differentially increased in cancerous cases compared to benign cases, while levels of succinate and citrate were reduced. Glutamate levels differentially increased in both biofluids while glutamine reduced in serum. Threonine and taurine levels depleted in cancer compared to benign, while increased trigonelline, tryptophan, and isoleucine levels were seen in malignancy compared to benign states. The levels of urinary glycine, creatinine and phenylalanine dropped in response to cancer development.

Urine and serum samples were further analyzed for their lipid content and association with disease using GCMS. There were no significant fatty acids detected in urine samples. However, several significant fatty acids were found in serum (Supplementary Table S1). Regression coefficient of variation plots (Supplementary Figure S4 and S5) were calculated for all of the NMR and GCMS models presented to show the most differential metabolites between the groups compared and potential biomarkers of interest.

### 2.6. Internal Validation and AUC

Receiver Operating Characteristic (ROC)curves were constructed for all GCMS and NMR models using Metz-ROC (University of Chicago, Chicago, IL, USA) (Supplementary Figure S3.) except for the pT1 versus pT3 GCMS model, which comprise of two diseased groups. The serum and urine GCMS models had AUC values of 0.93 and 0.98, respectively, and AUC values for NMR models ranged from 0.83 to 0.98.

## 3. Discussion

Most patients with RCC are diagnosed incidentally and metabolomics presents a platform that may potentially allow for a non-invasive means to discriminate between benign and malignant lesions [25]. In general, metabolomics relies on a “biopattern” representing a set of metabolites that is influenced by the disease in a specific and coordinated manner rather than a single metabolite. This comprehensive approach can be achieved by combining data obtained through complementary analytical platforms, such as ^1^H NMR spectroscopy and various forms of mass spectrometry, e.g., GCMS or LC-MS.

In this study, we have evaluated the feasibility of serum and urine metabolomics for identifying malignant renal masses and distinguishing stages of RCC. Overall, we found that NMR and GCMS coupled with multivariate statistical analysis (OPLS-DA) can distinguish between malignant and benign renal masses with up to 98% specificity and sensitivity. We also found that integrating NMR and GCMS datasets revealed better discriminatory power and higher predictive ability. The discriminatory power of GCMS and multivariate statistical analysis and their application to RCC urine analysis has been demonstrated in other studies as well [26].

Our study gives a comprehensive overview of the metabolic signature of RCC biofluids. It is well known that RCC features a metabolic shift towards aerobic glycolysis (Warburg effect) [27,28], and this is mirrored in our study by decreased levels of Krebs’s cycle intermediates: citrate and succinate and increased levels of glycolytic products; pyruvate and lactate in RCC samples relative to controls. While some studies have obtained similar results with comparison between healthy controls and RCC samples [8,9,13], our novel findings demonstrate interesting metabolic differences between benign and cancer cases.

Lactate levels particularly increased significantly in these studies [9,29], this may be indicative of increased glycolytic activity and inefficient production of ATP via glucose shunt to lactate rather than through the TCA cycle. This characteristic inadvertently results in decreased levels of TCA cycle intermediates: citrate and succinate. Deregulation of these metabolites is common in other types of cancer [30,31] and has previously been reported in RCC [10,32]. A truncated Kreb’s cycle seems to be a metabolic feature that is associated with metabolic derangement even in non-malignant type 2 diabetes cases [33].

Given that 70%–90% of clear cell RCC cases (the majority histology type in this study) are found to be associated with loss of the von Hippel Lindau (VHL) gene, and, consequently, an activation of HIF, the resulting metabolic profile is to be expected [34]. Specifically, the role of HIF in the regulation of cellular glucose flux and shunting of pyruvate from the TCA cycle towards lactate production in renal cancer cells becomes apparent. The inactivation or loss of the VHL tumour suppressor is the main molecular trigger for altered metabolism in ccRCC. Protein interaction studies revealed that VHL belongs to the E3 ubiquitin family of ligases and forms a stable complex with elongin B, elongin C and cullin 2 [34]. In normoxia, the VHL complex binds the hydroxylated α-subunit of HIF-1, thereby labelling it for proteasomal degradation. With the loss of VHL in ccRCC, hydroxylated HIF-α “escapes” degradation, becomes stabilized and translocates to the nucleus where it dimerizes with HIF-β. The HIF-1 complex then binds the hypoxia response element (HRE) motif of target genes to induce or repress transcription [35]. The result of this process includes the regulation of glucose transporters and expression of glycolytic enzymes, regulation of oxidative phosphorylation via TCA cycle and lipogenesis in VHL-lacking RCC patients [36,37].

Furthermore, the role of substitute glucose utilization pathways including pentose phosphate pathway in the tumorigenesis of RCC has been elucidated, and it is suggested that ccRCC reprograms the cells energy metabolism for biomolecule assemblage by diverting metabolic intermediates for anabolic purposes [38], and this phenomenon becomes more apparent as the disease progresses [10]. Cross-platform molecular analyses of mRNA, miRNA, DNA methylation and protein conducted on ccRCC nephrectomy samples have shown that alteration in molecular metabolism, which stem from the diversion of intermediates towards pentose phosphate pathway, downregulated Krebs cycle enzymes and reduced AMPK along with the upregulation of glutamine flux was associated with unfavorable prognostic outcome in ccRCC patients [39].

Differential lipid metabolites identified in our serum GCMS results (Supplementary Table S1) further highlight the role of altered metabolism in RCC. Like the forms of metabolic deviations mentioned above, altered fatty acid metabolism seems to be associated with higher grade RCC, which is a pointer to the shift in energy reliance from glycolysis to other sources as the disease progresses. Neoplastic cells are known to satisfy their high metabolic needs through mechanisms that include fatty acid breakdown and other non-glycolytic metabolism [13] and acquire most of their fatty acid quota from de novo synthesis. Hence, altered lipogenesis is characteristic of cancer [40,41].

There is overwhelming evidence that supports the dependence of cancer cells on the glutamine/glutamate pathway [42,43]. Glutamine is an alternative source of energy for living cells which is converted to glutamate and fed into the TCA cycle via α-ketoglutarate for energy and biomass production [44], most dividing cells in turn utilize glutamate in nucleotide synthesis.

In the present study, we considered the apparent alterations in glutamate and glutamine concentration of our samples. We recorded an increase in glutamate levels in RCC cases relative to controls, which corroborates findings in similar studies [8,10] and further strengthens the proposition that increased glutamate levels may indicate increased glutaminolysis for biosynthetic purposes in RCC. Taken together, this sequence of biological events further re-iterates the role and importance of metabolic remodeling in RCC tumorigenesis. Hence, therapeutic alternatives focused on extenuating these biological loopholes may improve the clinical outcome of RCC patients.

Increased levels of trigonelline and reduced 3-hydroxybutyrate in patient urine may be associated with smoking in these individuals. This connection has been previously reported in an unmatched RCC patient cohort study that exposed possible confounders [16].

The AUC values calculated in this study exceeded 0.8. This indicates excellent predictive ability of these metabolomics platforms and shows that they can reliably distinguish between benign and malignant renal masses and identify different stages of RCC. The AUC values computed for the GCMS analysis are considerably higher than the NMR values, which may be because of the higher sensitivity associated with GCMS. The urine samples also seem to be better predictors of RCC stages than the serum samples, especially in the integrated dataset where both R2 and Q2 metrics and AUC were substantially improved.

In this preliminary study, fatty acid assessment of RCC samples also provided insight into the influence of lipid metabolites on the disease. While there was no significant fatty acid detected in the urine samples, most of the statistically significant ones found in serum are of food origin and are linked to the fatty acid biosynthetic pathway and beta-oxidation of fatty acid, which may be implicated in energy production and cell membrane synthesis, as well as cell signaling and growth processes that are crucial to tumor progression.

This study is limited by a small sample size and the lack of an external validation cohort at the time of the study. The internal cross-validation and permutation tests were helpful in validating the OPLS-DA results; this intriguing initial observation, however, requires external validation. Hence, it is imperative that further studies be conducted to replicate these results using a larger and more diverse patient cohort.

## 4. Materials and Methods

### 4.1. Study Population

This study received approval by our institutional review board, and study participants signed an informed consent. Blood samples were drawn from fasting patients the morning of surgery and collected in buffer free containers. After centrifugation, the serum was stored at −80 °C until the time of analysis. Urine samples were obtained from fasting patients immediately prior to surgical removal of enhancing masses suspected for RCC. Benign lesions were determined by post-operative pathology and compared to pathologically confirmed RCC.

### 4.2. ^1^H NMR Analysis

Samples from both groups, 75% of which are of clear cell (ccRCC) histology type were prepared and analyzed according to a previously described protocol with minor modifications [45]. Briefly, 53 serum and urine samples (200 μL each) were thawed on ice and filtered in prewashed 3 kDa NanoSep microcentrifuge filters to remove protein and other large impurities. Protein filtered from serum samples were washed with an additional 100 μL of D_2_O. The filtrates (volume ranging from 100–150 μL) were then transferred to clean microcentrifuge tubes and the final volume brought to 400 μL by the addition of 80 μL of phosphate buffer (0.5 M NaH_2_PO_4_ buffer solution at pH 7.0) containing 2.5 mM 2,2-dimethyl-2- silapentane-5-sulfonate (DSS, final concentration 0.5 mM), 10 μL of 1M sodium azide to prevent bacterial growth, and D2O. The final concentration of DSS (0.5 mM for each sample) was used for NMR chemical shift reference and concentration calibration. The pH of each sample was adjusted to 7.00 ± 0.04. Untargeted one-dimensional proton ^1^H NMR analysis was carried out on a Bruker Avance 600 NMR spectrometer (Milton, ON, Canada) operating at 600.22 MHz and 298 K and equipped with a 5-mm TXI probe and an automated NMR case sample changer. NMR data collection measured all detectable compounds between 0–10 ppm and was devoid of any metabolite pre-selection. To confirm chemical shift assignments, two-dimensional NMR experiments including Total Correlated Spectroscopy (2D ^1^H–^1^H TOCSY) and Heteronuclear Single Quantum Correlation (2D ^1^H–^13^C HSQC) were performed on the last two samples of every batch. High resolution ^1^H NMR spectra were collected using a standard Bruker pulse sequence program (pr1d_noesy) that features water suppression, with 1024 transient acquisitions, and these spectra recorded from 0–10 ppm were processed using the TopSpin software (Bruker, ON, Canada).

Using the Processor module of the Chenomx NMR Suite 7.5 software (Chenomx Inc. Edmonton, Canada), a line broadening of 0.2 Hz was applied to all spectra followed by phase correction, water region deletion, baseline correction and reference deconvolution with DSS peak calibration. Using the Chenomx Profiler module, the NMR peaks were assigned to their corresponding metabolites and quantified in a “targeted” approach where about 95% of the peaks in the serum spectrum (about 60% in urine) were accounted for [46]. We only used the metabolites that were confidently identified and quantified by the Chenomx Profiler for further analyses, to better focus on the parts of the metabolomic profile whose biological significance and roles have been previously described.

The Chenomx Suite is equipped with reference libraries that contain numerous pH-sensitive compound models that are identical to the spectra of pure compounds obtained under similar experimental conditions. The underlying spectral libraries have been developed using a combination of mathematical algorithms and actual NMR measurements collected at a variety of pH conditions. During targeted profiling, these models are automatically calibrated by adjusting compound line shapes, peak widths, and chemical shifts, to better match the sample conditions. 

Variation between metabolites’ concentrations warranted spectral normalization using the Probabilistic Quotient Normalization (PQN) approach where the calculated median of all samples was used as the reference spectrum [47]. This normalized dataset was then used for further analyses.

### 4.3. GCMS Analysis

Metabolite extraction was performed according to previously described methods [48]. A biphasic mixture of chloroform and methanol was added to each sample and the separated aqueous fraction was vacuum-dried (SpeedVac, Eppendorf, Germany) and stored at −20 °C pending derivatization. The dried aqueous samples were then re-suspended in 50 µL of 20 mg/mL Methoxylamine-hydrochloride/pyridine at 37 °C for 2.5 h during derivatization and a silylating agent; N-Methyl-N-(trimethylsilyl) trifluoroacetamide (MSTFA; Sigma-Aldrich, Oakville, ON, Canada) was added. Each sample was diluted with 500 µL of Hexane containing (1 ug naphthalene-d8/ml) and centrifuged at 13,200 rpm (Eppendorf 5415) for 4 minutes to remove particles that may interfere with subsequent analyses. GCMS vials with glass inserts filled with 200 µL of sample supernatant before insertion into the Waters GCT mass spectrometer-coupled Agilent chromatograph 7890A (Agilent Technologies Canada Inc, Mississauga, Ontario, Canada) equipment, which employs the GC-TOF-MS procedure for analysis. Mass spectra of the aqueous and fatty acid fractions were processed using Metabolite Detector software (version 2.1N-2012-03-02, Technische Universität Carolo-Wilhelmina zu Braunschweig, Braunschweig, Germany) and MET-IDEA (version 2.08 2012-05-03, The Samuel Roberts Noble Foundation, Inc., Ardmore, OK, USA), respectively. The Golm metabolome database (GMD) was used for metabolite identification [48]. Compounds in the aqueous fraction were normalized by the PQN method, after features with more than 50% relative standard deviation (RSD) were removed [47]. All compounds were used for further analysis using an untargeted and comparative approach. 

### 4.4. Statistical Analysis

Normalized NMR and GCMS data were imported to SIMCA-P+ 12.0.1 software (Umetrics, Umea, Sweden) where log transformation, centering and unit variance scaling were performed. Individual and integrated (combined NMR and GCMS data) analyses of the data sets were also performed, and the mean values of metabolites common to both analytic platforms were calculated and included in the integrated dataset. Models of unsupervised principal component analysis (PCA) were initially constructed to identify potential outliers and groups of observations that may form distinct patterns. Using supervised orthogonal partial least squares-discriminant analysis (OPLS-DA), statistical models, in which two of the three groups were compared per time, were built. R2Y and Q2 metrics which describe the explained variation within the data set and the predictability of the model, respectively, were calculated based on the averages of the sevenfold cross-validation. R2Y and Q2 values range between 0–1, and the closer these metrics are to 1, the higher the variance explained by the model and the more reliable the predictive power of the model. PLS-DA models were built for each comparison and were found to have metrics similar to their OPLS-DA counterparts. The problem of OPLS-DA analysis being prone to overfitting is therefore ruled out in this dataset. Furthermore, Variable Influence on Projection (VIP) values of OPLS-DA models were used to extract the most influential metabolites contributing to group separation, and only metabolites with VIP scores > 1 were considered potentially relevant. Statistically significant metabolites were further determined by Student paired *t*-test (*p* > 0.05). A 999 times permutation test assessed the validity and non-randomness of the OPLS-DA models [49]. The permutations Q2 intercepts that were calculated are shown alongside the model statistics in Table 2. The Q2 intercept validates the reproducibility and robustness of a model and the more negative the value, the better the validity of the model, as it is an indication that the model metrics get progressively worse with every permutation introducing mislabeled variables [50,51]. The relationship between the groups and the differential metabolites are shown in regression coefficients of variation plots (Supplementary Figures S4 and S5).

Metz-ROC (University of Chicago, City, IL, USA) was used to calculate AUC. The specificity and sensitivity were calculated for sample class predictions made by a 7-fold cross-validation process (Y-predcv, predictive Y variables; SIMCA-P+ software). In this internal validation method, the data are divided into 7 portions and each 1/7th is sequentially excluded until all data is analyzed. The average predicted group values were used to create receiver-operating characteristic (ROC) curves for each comparison and to calculate the area under the curve (AUC).

## 5. Conclusions

In conclusion, serum and urine based metabolomics can distinguish RCC from benign renal masses, as well as pT1 from pT3 RCC. These tools can be potentially employed clinically to identify renal neoplastic transformation in asymptomatic individuals.

## Figures and Tables

**Table 1:** Clinicopathologic characteristics of 53 patients with renal masses undergoing metabolomics analysis. RCC: Renal cell carcinoma. BMI: Body Mass Index.

Sample Group	Number of Sample (Urine and Serum)	Age at Surgery Range (Years)	Mean Age (Years)	Number of Men	Number of Women
Controls	13	39–69	53.67	9	4
All RCC	40	36–84	61.89	23	17
ccRCC	37	36–84	62.49	22	15
Papillary	2	37–72	53.91	1	1
Unclassified	1	56	55.52	-	1
Stage I (T1a: 22; T1b: 6)	28	36–84	60.88	16	12
Stage II	1	46	45.52	1	-
Stage III	10	59–80	66.35	6	4
Stage IV	1	57	56.87	-	1
Smokers	20	50–84	62.91	14	6
Non-smokers	28	36–82	57.31	13	15
Unknown smoking status	5	62-69	65.30	4	1
BMI 19–25	13	39–84	61.02	4	9
BMI above 25	28	36–74	58.02	21	7
BMI unknown	12	45–80	63.94	7	5

**Table 2:** Statistical metrics of individually analyzed and Integrated NMR and GCMS orthogonal partial least squares discriminant analysis (OPLS-DA) models (B = Benign).

Model Type	R2	Q2	CV Anova *p*-Value	Q2 Intercept
NMR serum				
B vs. pT1	0.46	0.28	3. 4 × 10^−3^	−0.27
B vs. pT3	0.58	0.37	1.6 × 10^−2^	−0.32
FuhrmanL vs. FuhrmanH	0.37	0.23	8.7 × 10^−3^	−0.24
NMR urine				
B vs. pT1	0.50	0.37	4.9 × 10^−4^	−0.28
B vs. pT3	0.72	0.68	2.6 × 10^−5^	−0.41
FuhrmanL vs. FuhrmanH	0.54	0.36	8.6 × 10^−4^	−0.29
GCMS serum				
B vs. pT1	0.63	0.48	3.2 × 10^−3^	−0.28
pT1 vs. pT3	0.70	0.54	4.1 × 10^−5^	−0.45
FuhrmanL vs. FuhrmanH	0.60	0.47	1.0 × 10^−5^	−0.26
GCMS urine				
B vs. pT3	0.87	0.70	3.4 × 10^−4^	−0.43
B vs. FuhrmanH	0.84	0.62	1.1 × 10^−3^	−0.46

**Table 3:** Differential metabolites linked to cancer in ^1^H NMR and GCMS analysis of serum and urine (significant metabolites are shown with *p* < 0.05).

Metabolomics Platform	Decrease in Cancer vs. Benign				Increase in Cancer vs. Benign			
	Serum	*p*-Value (<0.05)	Urine	*p*-Value (<0.05)	Serum	*p*-Value (<0.05)	Urine	*p*-Value (<0.05)
^1^H NMR Metabolites	Citrate		Citrate	0.039	2-oxoisocaproate		Pyruvate	0.040
	Methanol		Succinate	0.004			Lactate	0.049
	Threonine Glycine		Glycine	0.047	Creatine		Oxypurinol	
	Histidine Taurine		3-hydroxybutyrate Creatinine		Isoleucine	0.041	Gluconate Hypoxanthine	
	Glutamine		2-aminoisobutyrate	0.008	Glutamate	0.003	Malonate	
			Phenylalanine	0.025	Ornithine	0.048	Betaine	
							Tryptophan	
			Methylhistidine	0.017			Trigonelline	
							Dimethylamine	
GCMS Metabolites	5-methylcytosine	0.049	Acetate Threonine		Glutamate Tyrosine		Glucose	0.001
	Eicosanoate	0.003	Gluconate		Octadecanoate		Erythritol	0.011
			Thymine		Galactose	0.030	2-oxoglutarate	0.032
			Mannitol		Pyruvate	0.018	Myo-inositol	0.040
			Citrate		Lactate	0.018		

## Supplementary Materials

The following are available online at www.mdpi.com/2218-1989/7/1/6/s1, Table S1. Fatty acids detected in serum RCC samples analyzed by GCMS; Figure S1. Characteristic NMR spectra of serum samples obtained from (A) benign (control) (B) RCC patients 0: 2-hydroxybutyrate, 1: leucine, 2: valine, 3: 3-hydroxybutyrate, 4: lactate, 5: alanine, 6: lysine (merged with DSS) 7: lysine, 8: glutamine, 9: citrate, 10: DSS, 11: lysine 12: glucose. 13: creatinine, 14: lactate, 15: glucose, *—deleted water region, 16: glucose, 17, 19: tyrosine, 18, 21: histidine, 20: phenylalanine; Figure S2. Integrated NMR and GCMS orthogonal partial least squares discriminant analysis (OPLS-DA) models distinguishing between benign and cancer cases. OPLS-DA score scatter plots showing separation in the serum and urine metabolic profile of (A) benign versus stage 1 cancer cases (B) benign versus stage 3 cancer cases (C) benign versus stage 1 cancer cases (D) benign versus stage 3 cancer cases, respectively, along their orthogonal partial least squares (OPLS1) and partial least squares components (PLS1); Figure S3. ROC curves depicting the predictive ability of the constructed models in each group comparison in serum and urine. (A) ROC curves illustrating the performance of the GCMS models in distinguishing between benign and stage 3 disease. (B) ROC curve illustrating the performance of the NMR models in distinguishing between benign and stage 3 disease. (C) ROC curve for the NMR models distinguishing between benign and stage 1 disease. AUROC–area under the ROC curve; TPF–true positive fraction (Sensitivity); FPF–false positive fraction (1–Specificity); Figure S4. OPLS-DA regression coefficient plot of NMR analyzed serum and urine. Positive coefficient values (upper portion of the plot) indicate increased serum metabolite concentrations in (A) stage 1 versus benign, (B) stage 3 versus benign, (C) Fuhrman high versus Fuhrman low; urine metabolite concentrations in (D) stage 1 versus benign, (E) stage 3 versus stage 1 (F) Fuhrman high versus Fuhrman low, while negative values (the lower part of diagram) show a decrease in metabolite concentrations; Figure S5. OPLS-DA regression coefficient plot of GCMS analyzed serum and urine. Positive coefficient values (upper portion of the plot) indicate increased serum metabolite concentrations in (A) stage 3 versus benign, (B) stage 3 versus stage 1, (C) Fuhrman high versus Fuhrman low; urine metabolite concentrations in (D) stage 3 versus benign, (E) Fuhrman high versus benign, while negative values (the lower part of diagram) show a decrease in metabolite concentrations.
