# Supplementary material for: Urine and Serum Metabolomics Analyses May Distinguish between Stages of Renal Cell Carcinoma
Source: Metabolites. 2017 Feb 3;7(1):6. doi: 10.3390/metabo7010006 (PMC5372209; doi:10.3390/metabo7010006)
Supplement: Supplementary file 1 [file metabolites-07-00006-s001.pdf]

Supplementary Materials: Urine and Serum Metabolomics Analyses May Distinguish between Stages of Renal Cell Carcinoma

Oluyemi S. Falegan, Mark W. Ball, Rustem A. Shaykhutdinov, Phillip M. Pieroraio, Farshad Farshidfar, Hans J. Vogel, Mohamad E. Allaf, Matthew E. Hyndman

Table S1. Fatty acids detected in serum RCC (renal cell carcinoma) samples analyzed by GCMS (gas chromatography-mass spectrometry).

	Serum		Urine
FATTY ACID	PATHWAY	MODEL	
NONE	-	B VS. T1	
PENTADECANOIC ACID	-		
PALMITIC ACID	As below	B VS. T3	
HEPTADECANOIC ACID	-		
DOCOSADIENOIC ACID	-	T1 VS. T3	NO SIGNIFICANT FATTY ACID FOUND
PALMITIC ACID	GLYCEROLIPID METABOLISM/function (signal transduction)		
LAURIC ACID	FATTY ACID BIOSYNTHESIS		
CAPRIC ACID	FATTY ACID BIOSYNTHESIS		
TRIDECANOIC ACID	-		

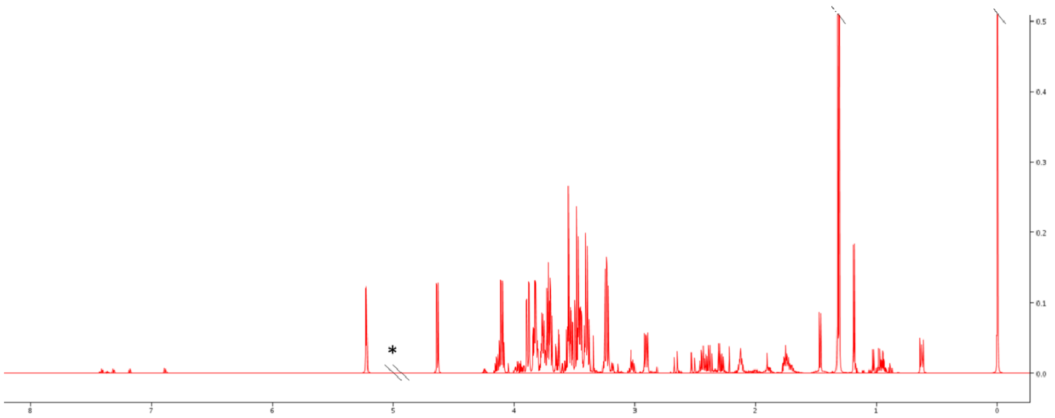

(A)

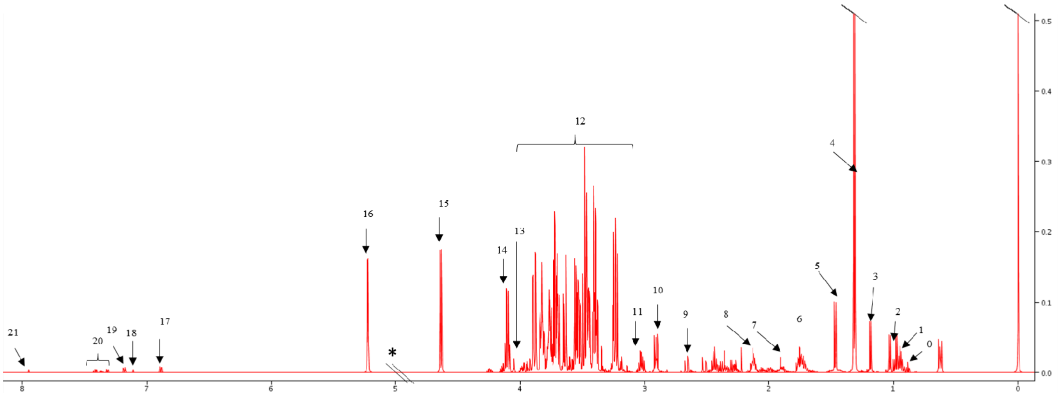

(B)

Figure S1. Characteristic NMR spectra of serum samples obtained from (**A**) benign (control); (**B**) RCC patients 0: 2-hydroxybutyrate, 1: leucine, 2: valine, 3: 3-hydroxybutyrate, 4: lactate, 5: alanine, 6: lysine (merged with 2,2-Dimethyl-2-silapentane-5-sulfonate (DSS)) 7: lysine, 8: glutamine, 9: citrate, 10: DSS, 11: lysine 12: glucose. 13: creatinine, 14: lactate, 15: glucose, *—deleted water region, 16: glucose, 17, 19: tyrosine, 18, 21: histidine, 20: phenylalanine.

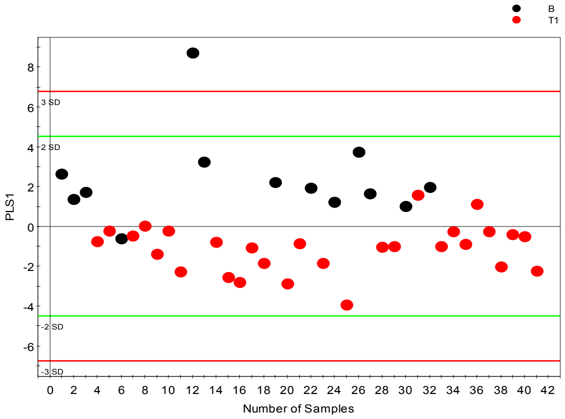

(A)

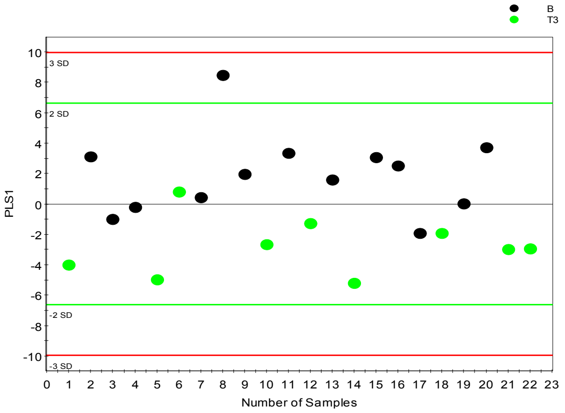

(B)

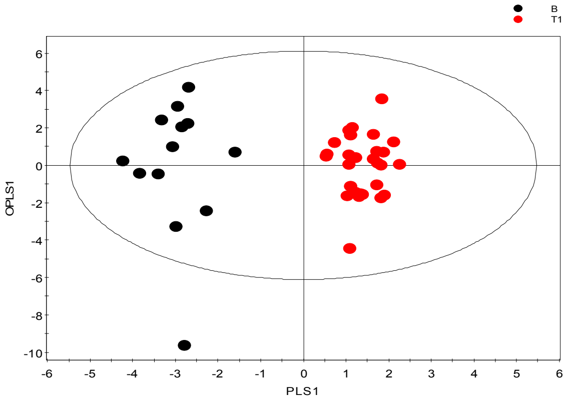

(C)

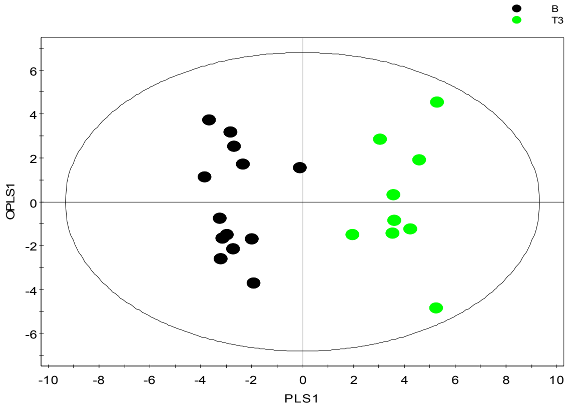

(D)

Figure S2. Integrated NMR and GCMS orthogonal partial least squares discriminant analysis (OPLS-DA) models distinguishing between benign and cancer cases. OPLS-DA score scatter plots showing separation in the serum and urine metabolic profile of (**A**) benign versus stage 1 cancer cases (**B**) benign versus stage 3 cancer cases (**C**) benign versus stage 1 cancer cases (**D**) benign versus stage 3 cancer cases, respectively, along their orthogonal partial least squares (OPLS1) and partial least squares components (PLS1).

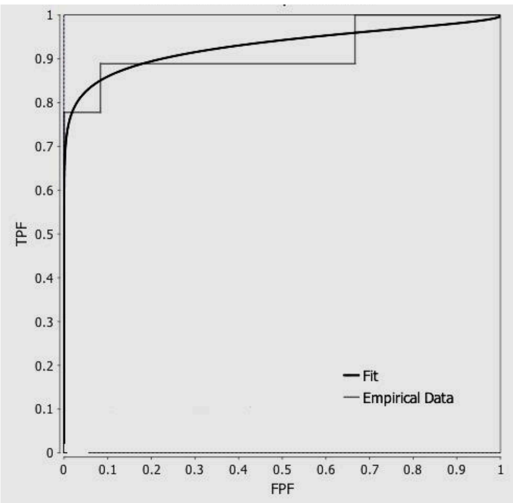

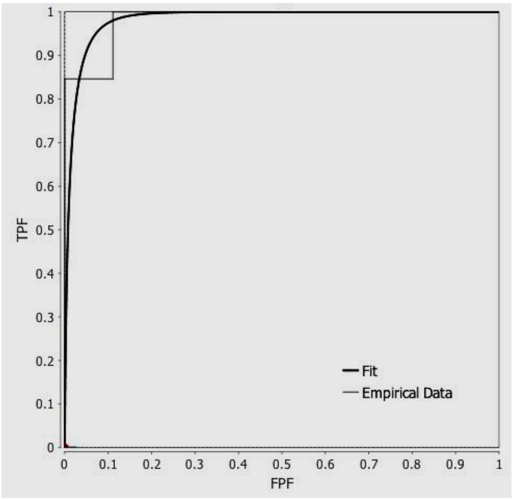

(A)

Figure 3. *Cont.*

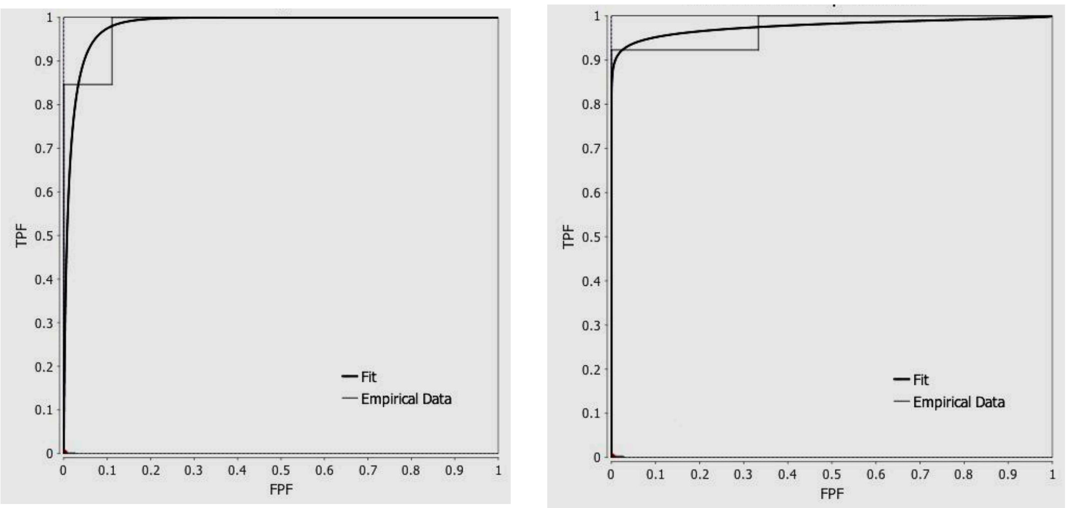

(**B**)

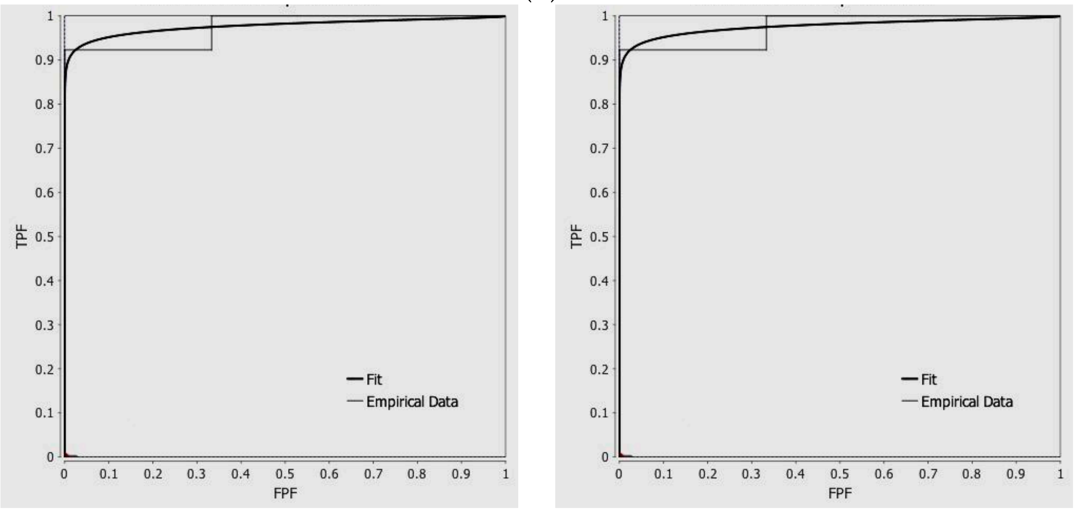

(**C**)

Figure S3. ROC curves depicting the predictive ability of the constructed models in each group comparison in serum and urine. (**A**) ROC curves illustrating the performance of the GCMS models in distinguishing between benign and stage 3 disease; (**B**) ROC curve illustrating the performance of the NMR models in distinguishing between benign and stage 3 disease; (**C**) ROC curve for the NMR models distinguishing between benign and stage 1 disease. AUROC—area under the ROC curve; TPF—true positive fraction (Sensitivity); FPF—false positive fraction (1—Specificity).

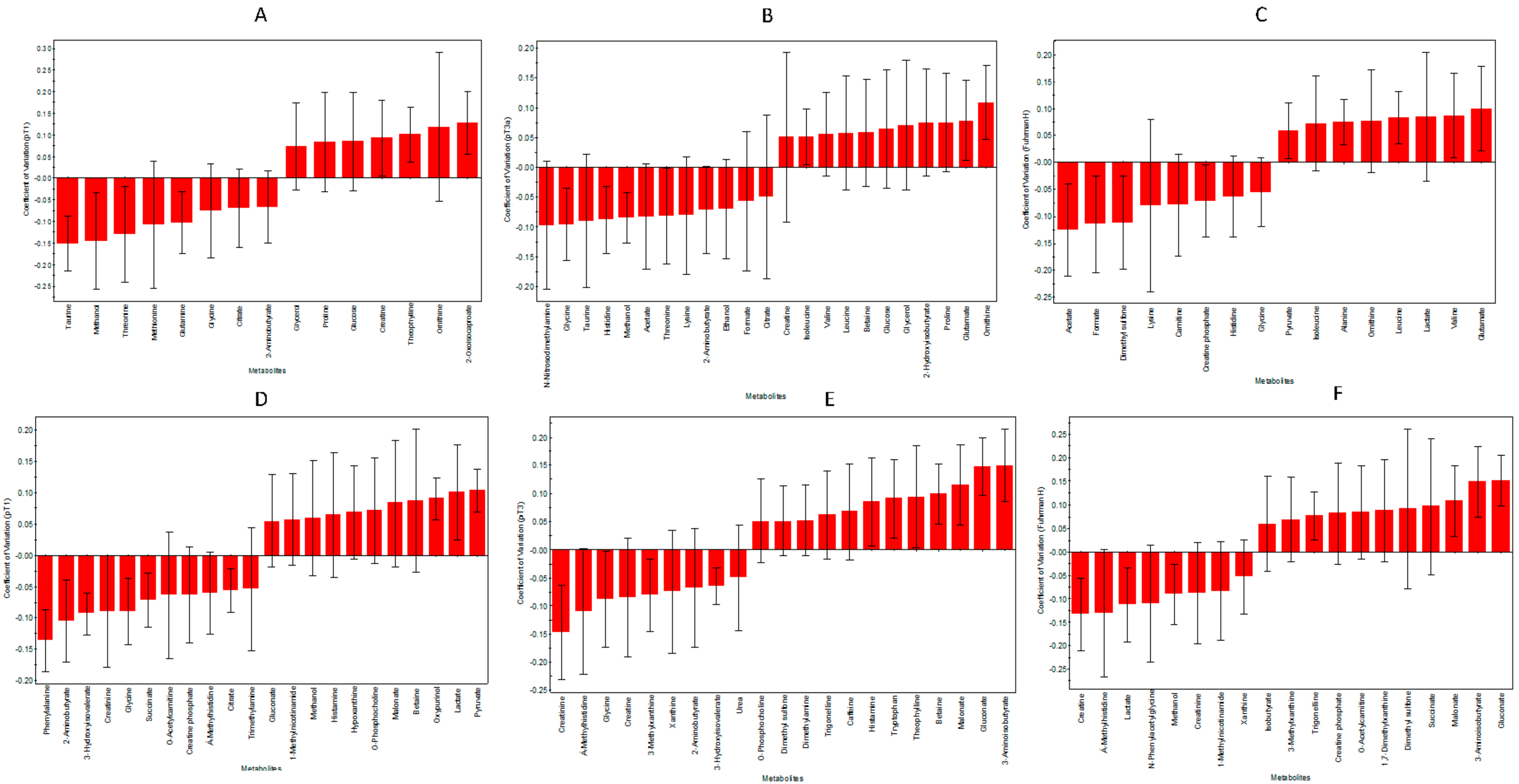

Figure S4. OPLS-DA regression coefficient plot of GCMS analysed serum and urine. Positive coefficient values (upper portion of the plot) indicate increased serum metabolite concentrations in (**A**) stage 1 versus benign; (**B**) stage 3 versus benign; (**C**) Fuhrman high versus Fuhrman low; urine metabolite concentrations in (**D**) stage 1 versus benign; (**E**) stage 3 versus stage 1; and (**F**) Fuhrman high versus Fuhrman low, while negative values (the lower part of diagram) show a decrease in metabolite concentrations.

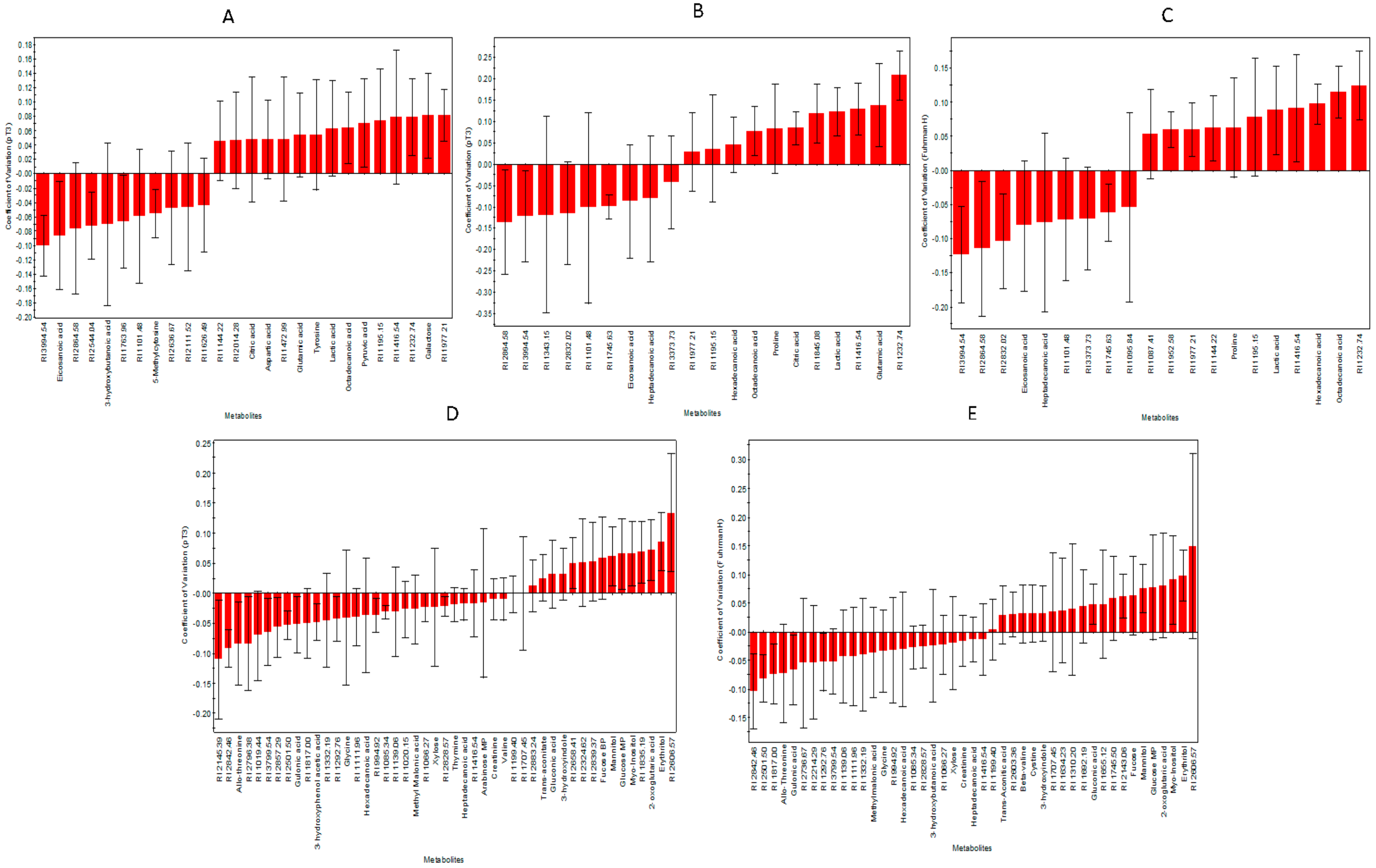

Figure S5. OPLS-DA regression coefficient plot of GCMS analysed serum and urine. Positive coefficient values (upper portion of the plot) indicate increased serum metabolite concentrations in (**A**) stage 3 versus benign; (**B**) stage 3 versus stage 1; (**C**) Fuhrman high versus Fuhrman low; urine metabolite concentrations in (**D**); stage 3 versus benign (**E**); and Fuhrman high versus benign, while negative values (the lower part of diagram) show a decrease in metabolite concentrations.
